# Meeting daily nutrition requirements using the Eating Disorder Healthy Eating Tool

**DOI:** 10.1186/2050-2974-3-S1-P20

**Published:** 2015-11-23

**Authors:** Susan Hart, Claire Marnane, Caitlin McMaster, Angela Thomas

**Affiliations:** 1 Royal Prince Alfred Hospital, Sydney, Australia; 2 Wesley Hospital Eating Disorder Program, Brisbane, Australia; 3 The Children's Hospital Westmead, Sydney, Australia

## 

The Eating Disorder Healthy Eating tool was designed for use when delivering education to eating disorder patients on the components of a healthy diet. In previous research presented at the ANZAED conference in 2011, it was shown that 19 of 20 participants (95%) attending Derwent House, the RPA eating disorder day program endorsed the tool as being more effective than a general tool for recovery from an eating disorder. One reason given for the preference included increased relevance of content. Since 2011, the pyramid has been used at Derwent House not only as an education tool, but also as a menu-planning tool in order to meet the nutrition requirements of attendees. Using nutrition databases, NUTTAB and Food Works, the portion sizes of each item in each food group of the education tool, has been modelled, to show how a balanced diet can be achieved for underweight and normal weight patients. Modeling was completed for meat containing, vegetarian or vegan diets. A balanced diet has been defined as the number of portions of each food group required to meet the Nutrient Reference Values published by the NHMRC. Results of this food modelling will be presented.

